# Innovative Incorporation of Poly(3,4-ethylenedioxythiophene)-poly(styrenesulfonate) as Hole Carrier Transport Layer and as Anode for Organic Solar Cells Performance Improvement

**DOI:** 10.3390/polym12122808

**Published:** 2020-11-27

**Authors:** Leon Hamui, Maria Elena Sánchez-Vergara, Ricardo Corona-Sánchez, Omar Jiménez-Sandoval, Cecilio Álvarez-Toledano

**Affiliations:** 1 Facultad de Ingeniería, Universidad Anáhuac México, Avenida Universidad Anáhuac 46, Col. Lomas Anáhuac, Huixquilucan 52786, Estado de México, Mexico; leon.hamui@anahuac.mx; 2 Departamento de Química, Universidad Autónoma Metropolitana, Unidad Iztapalapa, San Rafael Atlixco 186, Col. Vicentina-Iztapalapa, Ciudad de México 09340, Mexico; rcorona@xanum.uam.mx; 3 Centro de Investigación y de Estudios Avanzados del Instituto Politécnico Nacional, Unidad Querétaro, Libramiento Norponiente 2000, Fracc. Real de Juriquilla, Querétaro 76230, Mexico; 4 Instituto de Química, Universidad Nacional Autónoma de México, Circuito Exterior S/N, Ciudad Universitaria, Ciudad de México 04510, Mexico; cecilio@unam.mx

**Keywords:** PEDOT:PSS, Fischer carbene, thin film, optical gap, electrical properties

## Abstract

In this work, we present a comparative study of benzoid poly(3,4-ethylenedioxythiophene)-poly(styrenesulfonate) (PEDOT:PSS) as electrode and as hole carrier transport layer (HTL) in the manufacture of organic photovoltaic devices using Fischer metal-carbene complexes. The performance of the different devices was evaluated for solar cell applications. Scanning electronic microscopy (SEM) and X-ray diffraction (XRD) were used to characterize the thin films that integrated the devices. A more ordered and crystallized active film microstructure is observed when using benzoid PEDOT:PSS as nucleation layer. The optical gap for both direct and indirect electronic transitions was evaluated from ultraviolet-visible spectroscopy data (UV-vis), as well as the absorption coefficient (α), and the values are in the range of 2.10–2.93 eV. Photovoltaic devices with conventional architecture, using two different chromium carbenes as active layers, were manufactured, and their electrical behavior was studied. The devices were irradiated with different wavelengths between the infrared and ultraviolet regions of the electromagnetic spectrum. Using the PEDOT:PSS film as hole carrier transport layer (HTL) decreases the slope on the ohmic and space charge limited current (SCLC) regions and eliminates the trap-charge limited current (T-CLC) mechanism. Furthermore, a saturation current of ~1.95 × 10^−10^ A and higher current values ~1.75 × 10^−2^ A at 4 V, ~4 orders in magnitude larger were observed. The PEDOT:PSS films as HTL in the devices reduced the injection barrier, thus showing a better performance than as anodes in this type of organic solar cells.

## 1. Introduction

A photovoltaic cell is a device that converts sunlight into electricity using semiconductor materials [1]. Organic semiconductors show great potential for photoconversion, on the basis of their delocalized π electron systems that can absorb sunlight, create photogenerated charge carriers and transport such charge carriers [2]. Additionally, organic semiconductors may show high absorption coefficients, which make them good chromophores for optoelectronic applications [2,3]. Another important optoelectronic characteristic in organic semiconductors is the fact that their electronic band gap can be engineered by chemical synthesis [2]. Group 6 Fischer metal-carbene complexes are an example of the above; depending on the metal, the functional group or the substituent found in the molecular structure is the charge transfer capacity and in general, the optoelectronic properties that the carbene presents [4,5,6,7,8,9,10,11,12,13,14]. Organic semiconductors can be classified into two classes: molecular semiconductors (MSCs), like the Fischer metal-carbene complexes, and π-conjugated polymers [14]. Both types of organic semiconductors can be processed as good quality thin films.

The efficient charge transport through a semiconductor film requires that such a film be continuous and ordered. In the case of films processed in solution, the crystallinity degree obtained is usually not comparable to that of vapor phase deposited films; amorphous domains giving place to lower charge mobility values are formed [15], so the quality of films processed in solution has a great dependence on the solubility of the semiconductor. In the case of π-conjugated polymers, it is possible to use dopants soluble in organic solvents in order to induce their solubility [16]. An example is the poly(3,4-ethylenedioxythiophene)-poly(styrenesulfonate) (PEDOT:PSS) system that exhibits solubility as a function of PSS, that acts as dopant, and it favors the PEDOT dispersion [17]. The PEDOT:PSS combination results in a water-soluble polyelectrolyte system with good film forming properties, high conductivity, high visible light transmission and excellent stability [18]. PEDOT:PSS films can be heated in air at 100 °C for over 1000 h, with only a minimal change in its conductivity. Although initially used as an antistatic coating in photographic films, several new applications for PEDOT:PSS have been implemented over the past few years, for example, as an electrode material in capacitors, as a material for through-hole plating of printed circuit boards and others are expected [19,20,21,22,23]. It is also very attractive for optoelectronic applications and photovoltaic devices due to its excellent mechanical flexibility, thermal stability, optic transparency in the visible range and high electrical conductivity [23].

When developing optoelectronics devices, glass is a very commonly used substrate, but by itself, it is an electric insulator. Therefore, a conductive material is usually deposited on top to act as an electric contact for the developed device. The most widely used material for thin film devices is indium tin oxide (ITO). It is important to mention that due to the high-cost indium shortage, its limited mechanical flexibility and the cracks that readily occur when the substrate is bent, it is necessary to develop intrinsically conductive polymers (ICPs) with both high transparency and high conductivity for flexible optoelectronics [24,25,26,27,28]. PEDOT:PSS has proven to be a reasonable substitute as an electric contact layer [28]; it can be used as electrode or combined with other electronic materials to facilitate the hole injection/extraction [2,29,30]. The PEDOT:PSS system has been mainly studied as anode in photovoltaic devices, but its behavior as hole carrier transport layer (HTL) has been little explored in organic solar cells [16,23,30,31,32,33,34,35]. The latter is because a great number of hole carrier transport layers have been investigated, and their selection, design and successful application depend mainly on the active material’s highest occupied molecular orbital (HOMO) and lowest unoccupied molecular orbital (LUMO) energies [36]. Moreover, PEDOT:PSS provides a smooth surface for film deposition, which is desirable to reduce current leakage. It is important to consider that studies carried out in PEDOT:PSS indicate that for specific applications such as gas sensors, thermo-electric or photovoltaic devices, the electrical conductivity of the polymeric system must be increased in the first place. This increase may be done following two main routes: (i) addition of dopants [33,37,38] and (ii) treatment with substances such as hydrazine [39], dimethyl sulfoxide [23], polar compounds such as ethylene glycol, erythritol, nitroethanol [24] and isopropanol [39], among others, which in most cases, have the function of changing PEDOT from the benzoid to the quinoid form. The objective of the present work is to manufacture and characterize photovoltaic devices using the PEDOT:PSS system as anode or as HTL, keeping PEDOT in its benzoid form, which even though is of lower conductivity, it is considered eco-friendly. The devices manufactured are aligned with transient electronics. Transient electronics represents an emerging class of technology whose key characteristic is that it physically disappears, in whole or in part, in a controlled fashion after it has served its targeted function [40,41]. In order to compare and determine the best performance of such PEDOT:PSS based devices, Fischer metal-carbene complexes were used as active materials, previously synthesized and studied as organic semiconductors by some of the authors of this work [13,14]. Two such metal-carbene complexes were used as thin films and their properties compared for their application in solar cell devices. These complexes do not require the use of aggressive solvents for their degradation and the compounds formed are salts with low environmental impact. The thin films used in the devices were morphologically and structurally characterized by scanning electronic microscopy (SEM) and X-ray diffraction (XRD). Their optical properties were evaluated by UV-vis spectroscopy and finally, the electric and photovoltaic behavior of the devices was evaluated by the collinear four tip method for different temperature and lighting conditions.

## 2. Materials and Methods

**Figure 1:**
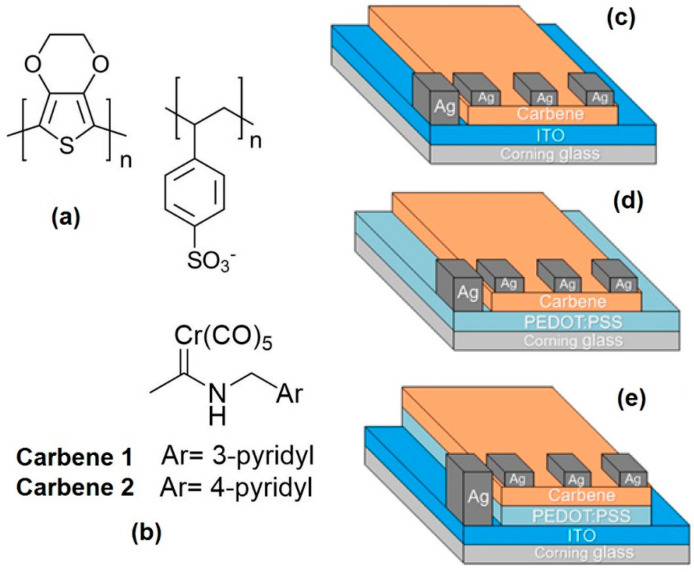
Structure of (**a**) poly(3,4-ethylenedioxythiophene)-poly(styrenesulfonate) (PEDOT:PSS) and (**b**) the Fischer metal-carbene complexes employed. Sandwich structure of the devices: (**c**) IC1 and IC2, (**d**) PC1 and PC2 and (**e**) IPC1 and IPC2.

A low conductivity grade 2.8 wt % poly(3,4-ethylenedioxythiophene)-poly(styrenesulfonate) (PEDOT:PSS) dispersion in H_2_O, was obtained from Sigma-Aldrich (Saint Louis, MO, USA) and required no further purification (Figure 1a). The Fischer metal-carbene complexes (Figure 1b) were synthesized and purified according to previous reports [13,14]. The optoelectronic devices were made by spin coating and high vacuum evaporation with the mentioned materials as thin films (Figure 1c–e). Three kinds of substrates were used to deposit the films: Corning glass slides, indium tin oxide (ITO) coated glass (Sigma-Aldrich, Saint Louis, MO, USA) slides and monocrystalline silicon (1 0 0) wafers. They all were subjected to ultrasonic cleaning and degreasing processes in different solvents, prior to their use. The PEDOT:PSS films were deposited by spin-coating, while the carbene films were made by high vacuum evaporation. The spin-coating technique was conducted with a Smart Coater 200 equipment (Laurell Technologies Corporation North Wales, PA, USA) set at an angular speed of 350 rpm, for 10 s. After deposit, the PEDOT:PSS thin films were heated at 80 °C for 90 s to carry out their polymerization. For the vacuum deposition process, an Intercovamex evaporation equipment (Intercovamex, S.A. de C.V., Cuernavaca, Morelos, México), with a tantalum boat, was used. The evaporation rate (4 Å/s), temperature (298 K) and pressure (1 × 10^−6^ Torr) in the vacuum chamber were maintained the same for all the deposition processes. The vacuum in the chamber was achieved by the operation of two pumps: a mechanical pump that generated an initial pressure of 5 × 10^−3^ Torr, and a turbo-molecular pump that allowed us to obtain a 1 × 10^−6^ Torr vacuum in the chamber. During the deposition processes, the films’ thicknesses were monitored with a quartz crystal monitor. For the characterization of the films, scanning electron microscopy (SEM) was conducted with a ZEISS EVO LS 10 scanning electron microscope (Zeiss International Inc., Göttingen, Germany), operated at a 20 kV voltage and a 25 mm focal distance, for films on Corning glass substrates. The X-ray diffraction analyses were performed on a Rigaku D-max 2100 diffractometer (Rigaku Corporation, Akishima-shi, Tokyo, Japan), with Cu *K*alpha1/*K*alpha2 radiation (1.5406/1.5444 Å), for films on glass substrates. The optical absorption for films on glass was also measured with a Unicam UV300 spectrophotometer (Thermo Fisher Scientific Inc., Waltham, MA, USA) in the wavelength range of 200–1100 nm. The current-voltage-luminance characterization was performed using a programmable voltage source, a sensing station with lighting and temperature controller circuits from Next Robotix (Comercializadora K Mox, S.A. de C.V., CDMX, Mexico) and an auto-ranging Keithley 4200-SCS-PK1 pico-ammeter (Tektronix Inc., Beaverton, OR, USA). Ag electrodes were painted on top of the samples on a four-point probe configuration to measure the electrical properties. The contacts were 2 mm wide and the distance between them was 1.6 mm; the thickness of the carbene 1 and carbene 2 films was 6.04 and 7.74 μm, respectively. The devices are divided into three groups, depending on their architecture (Figure 1c–e), named as IC, PC and IPC, where IC = Corning glass/ITO/carbene/Ag, PC = Corning glass/PEDOT:PSS/carbene/Ag and IPC = Corning glass/ITO/PEDOT:PSS/carbene/Ag. Each group consists of two similar sandwich structures, where the only difference is the type of carbene employed (Figure 1b). The latter gives the difference between IC1, PC1 and IPC1, where carbene 1 is used, and IC2, PC2 and IPC2, where carbene 2 is employed. Such sequences of layers were designed for the analysis of the effects of using ITO vs. PEDOT:PSS in combination with carbene organic semiconductors, as well as to examine the effects of using ITO as an electric contact and PEDOT:PSS on top of it as an HTL.

## 3. Results and Discussion

### 3.1. Films Deposition and Characterization

**Figure 2:**
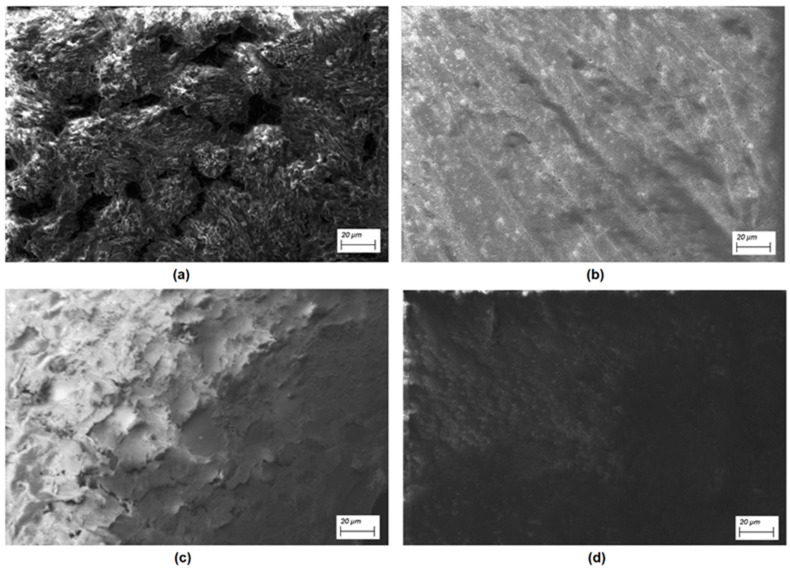
SEM images (500 ×) of the (**a**) carbene 1 and (**b**) carbene 2 films on ITO, and (**c**) carbene 1 and (**d**) carbene 2 films on PEDOT:PSS.

**Figure 3:**
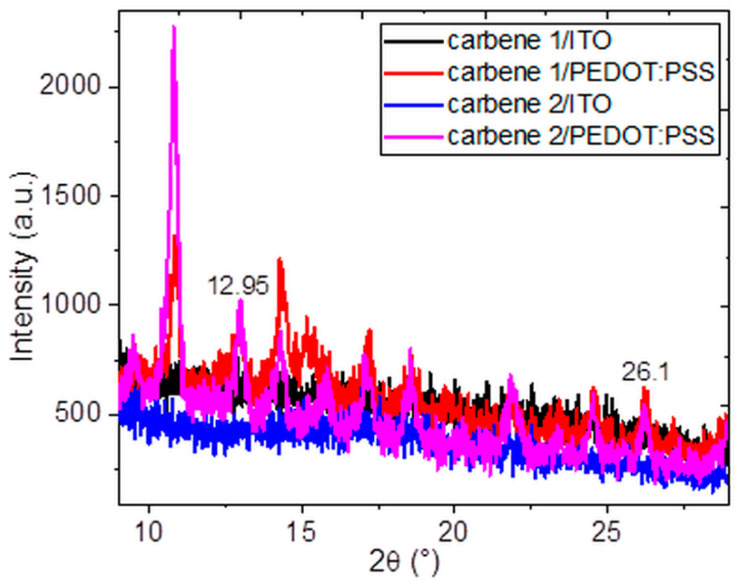
XRD patterns of the carbene films onto ITO and PEDOT:PSS.

In order to understand the conduction mechanisms, the morphology of the carbene films on the different substrates was first studied using SEM. Figure 2 shows the SEM images of the films deposited over ITO and PEDOT:PSS, at a 500 × magnification. Important differences in the morphology of the films are observed: in the case of the films directly deposited onto ITO (Figure 2a,b), there are hollows (carbene 1) and apparently a preferential direction of growth (carbene 2). Both films are heterogeneous and in the case of carbene 1, rounded structures of size superior to 10 μm were observed. On the other hand, the films deposited on PEDOT:PSS (Figure 2c,d) show a flake-like morphology [42], the films are more homogeneous and the number of hollows decreases with respect to the films deposited on ITO; the same occurs with the carbenes particle size. Apparently, when the deposits are made on PEDOT:PSS, the nucleation is greater, which generates a minor growth of these nuclei. Therefore, the films deposited on PEDOT:PSS generate a smoother surface. In order to complement the information regarding the structure of the films, XRD studies were performed. Figure 3 shows the XRD patterns obtained and apparently the films with PEDOT:PSS are more crystalline, although in general the films are mainly amorphous. The films onto PEDOT:PSS show a characteristic peak at nearly 2θ = 26°, which can be attributed to the interchain planar ring stacking [33,42,43,44]. Additionally, the diffraction peak with low intensity at approximately 2θ = 13° is related with the two-dimensional stacking arrangement of polymer chains in thin films [33,42,45]. A comparison of the two carbenes reveals that carbene 2 has a higher crystallinity degree than carbene 1. Apparently, PEDOT:PSS generates nucleation sites, promoting the carbene film to grow in a more orderly way, compared to its growth when it is deposited directly on the ITO. If the transport of charges is more efficient in films with ordered structures, a better electrical behavior can be expected in devices made with PEDOT:PSS films.

**Figure 4:**
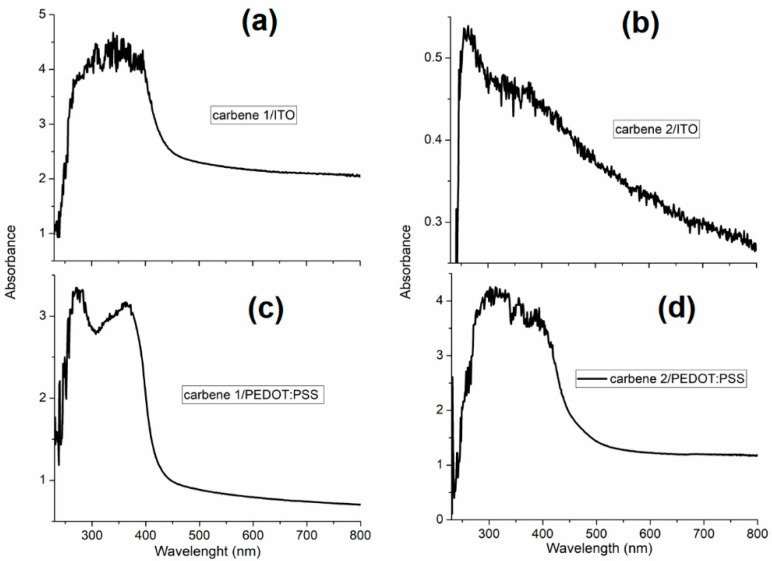
UV-vis spectra for the carbene films deposited on (**a**,**b**) ITO and (**c**,**d**) PEDOT:PSS.

Further information about the structure of the carbene films has been obtained by the analysis of their UV-vis spectra. In order to investigate the mechanism of conductivity, the analysis of chemical bonding states of the films was carried out using absorption spectroscopy [45]. Figure 4a,b shows the UV-vis spectra for the carbene films grown onto ITO and differences can be observed in the absorption spectra of the films: while carbene 1 shows a maximum at ~338 nm, thin films of carbene 2 show a maximum at ~261 nm. These peaks in the ultraviolet region of the spectrum correspond to the π–π* transitions between the different vibrational levels in the first singlet excited state and the ground state [14]. Figure 4c,d shows the UV-vis absorption spectra for the carbene films on PEDOT:PSS; two absorption peaks observed at ~275 nm and ~367 nm correspond to the aromatic rings of PSS [23,46]. From Figure 4, it is clear that the intensity of these two bands decreased for carbene 1, which implies that the amount of PSS chains in the polymer film is reduced with its presence [23,46,47]. This result demonstrates that the insulating PSS in the film was selectively removed due to the position of the substituent on the carbene and therefore carbene 1 on PEDOT:PSS should be the system that generates the greatest charge transport. The calculation of the optical activation energy or optical gap complements the previous results; it is known within the field of organic semiconductors that low gaps are particularly interesting in polymeric films, because of their high intrinsic conductivity in a neutral state and their transparency in a doped state [48]. It should be mentioned that the optical gap is the energy necessary for the photons to be absorbed and an exciton is formed (hole-electron pair linked through a coulombic interaction) inside the films [49]. In order to evaluate the optical gap of the films, the analysis of the UV-vis spectra was performed to obtain the absorption coefficient (α) and the photon energy (hν), and then Tauc’s method was applied [50,51,52]. This method consists of the extrapolation of the linear stretch of the skirt of the lower energy band of (αhv)^2^ and (αhv)^1/2^ (for direct and indirect electronic transitions, respectively) to the cutoff point with the abscissa axis corresponding to the energy of the photon (hν). Frequency (*ν*) is the inverse of the wavelength, the absorption coefficient (*α*) is obtained from the absorbance data, and *h* is Planck’s constant. The direct and indirect optical gap values obtained for each film are presented in Table 1. The distinction between direct and indirect gap concerns the relative position of the HOMO (highest occupied molecular orbital) and the LUMO (lowest unoccupied molecular orbital). In a direct gap material, both positions occur in the central zone, and during the transition the electron jumps from the HOMO to the LUMO by absorbing a photon. For an indirect gap material, on the other hand, it is not possible to perform the jump between the HOMO and LUMO by only absorbing a photon; the transition should involve a phonon to preserve the moment [53]. Table 1 shows that the optical gap values are in the range of 2.10–2.93 eV, the lowest values being for indirect transitions, which is expected considering the amorphous nature of the films. On the other hand, the carbene films deposited on PEDOT:PSS exhibit the lowest optical gap values, which is also expected, since according to SEM and XRD, their morphology is more homogeneous than that of the films deposited on ITO. However, the optical gaps of all the films are within the range established for organic semiconductors, and therefore, they may be used in photovoltaic devices.

### 3.2. Electrical Characterization

**Figure 5:**
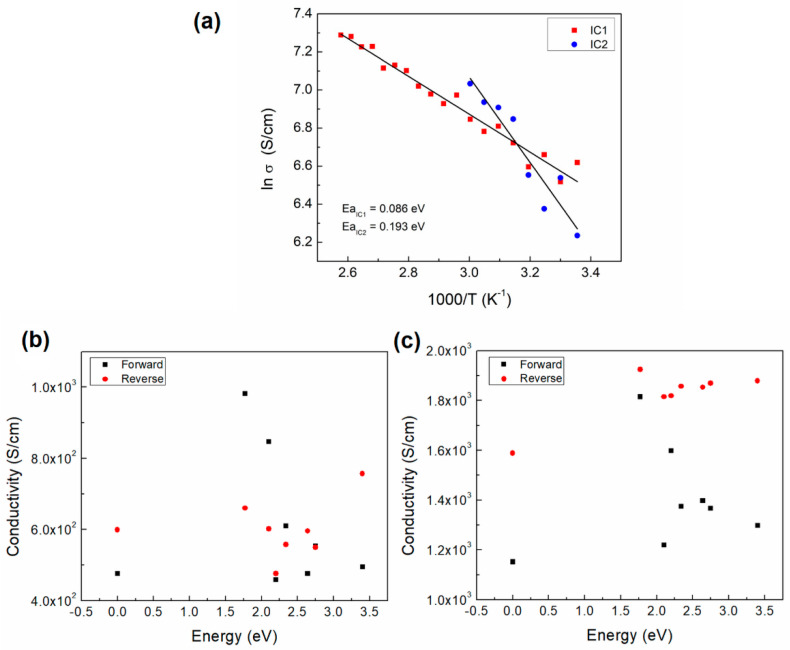
(**a**) Conductivity Arrhenius plot of IC devices. Conductivity vs. incident light energy of (**b**) IC1 and (**c**) IC2 devices.

We initially proceeded to study the electrical conductivity of carbenes without the polymer, namely, devices IC1 and IC2 (Figure 1c). The corresponding carbene conductivity measurements were carried out, and the temperature was gradually increased. These materials function as the active layer in the devices, so the electrical conductivity of the latter was expected to increase with temperature. Figure 5a shows the Arrhenius plot for the carbene 1 and carbene 2 devices. It is observed that while the temperature is increased, the conductivity for both devices also increases. It is important to notice a variation in the slope comparing both carbenes: it is steeper for the carbene 2 device. From the slopes in Figure 5a, a calculated activation energy of 0.086 eV was obtained for carbene 1, while a value of 0.193 eV resulted for carbene 2, more than 2 times greater than the first.

The ln of the conductivity values at room temperature are observed to be higher for carbene 1 (~6.6) than for carbene 2 (~6.2). Nevertheless, the carbene 2 conductivity becomes larger for temperatures higher than ~317 K, where both curves intercept at a ln(σ) value of ~6.7. The carbene devices conductivity for different light colors was obtained and plotted in Figure 5b,c. Figure 5b shows a variation of the conductivity of as much as ~5 × 10^2^ S/cm when varying the light on forward bias, and as much as ~3 × 10^2^ S/cm for reverse bias. The highest conductivity, 1 × 10^3^ S/cm, was observed for 1.8 eV on forward, while ~7.8 × 10^2^ S/cm was obtained on reverse bias. No clear tendency of the conductivity for carbene 1 was observed. For the IC2 device, Figure 5c shows a variation of the conductivity of as much as ~6.5 × 10^2^ S/cm when varying the light on forward bias, and as much as ~3.5 × 10^2^ S/cm for reverse bias. The highest conductivity, 1.8 × 10^3^ S/cm, was obtained for 1.8 eV on forward, while a value of ~1.95 × 10^3^ S/cm (1.8 eV) was found on reverse bias. The carbene 2 conductivity values are higher than those of carbene 1, even in dark conditions, where values of ~1.18 × 10^3^ S/cm and ~4.9 × 10^2^ S/cm, respectively, were observed. The latter could be related to the higher crystallinity exhibited by carbene 2. A defined trend of the conductivity for carbene 2 on forward bias is not observed, but it can be clearly seen that the reverse bias values are higher. In dark conditions, a conductivity increase between the forward and reverse bias values is observed for both devices, with a change of ~1 × 10^2^ S/cm (20%) and of ~4.2 × 10^2^ S/cm (36%) for carbene 1 and carbene 2, respectively. This change is related to the device breakdown, which increases the reverse current drastically. Thus, the reverse bias curve slope is more pronounced for the carbene 2 device.

**Figure 6:**
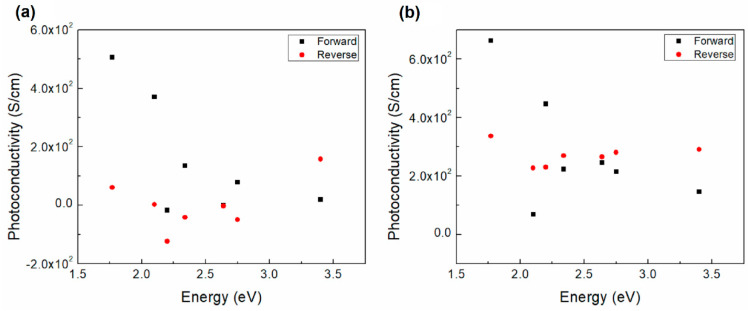
Photoconductivity vs. incident light energy of the (**a**) IC1 and (**b**) IC2 devices.

**Figure 7:**
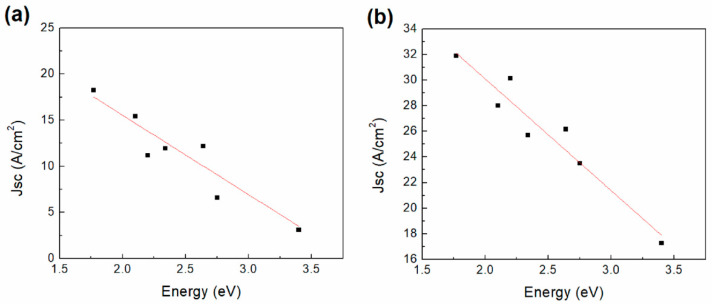
Jsc vs. incident light energy of the (**a**) IC1 and (**b**) IC2 devices.

To evaluate the photoresponse of the films, the photoconductivity was plotted against the incident light as shown in Figure 6. The photoconductivity behavior for both carbene devices on forward bias is very similar, showing an increase at ~1.75 eV and then a decrease to values of almost zero. The reverse bias does not show any specific tendency for carbene 1, while for the carbene 2 device it shows a slight increase for values larger than 2 eV. The highest photoconductivity was observed at 1.75 eV in Figure 6a (~5 × 10^2^ S/cm) and 6b (~6.8 × 10^2^ S/cm). The observed photoconductivity values are higher for carbene 2 than for carbene 1, which may be related to the smaller optical gap previously reported (Table 1). The short circuit current density (Jsc) plotted against the incident light energy is presented in Figure 7 for the carbene 1 and carbene 2 devices. A decrease of the Jsc is observed in both cases as the light energy increases. The corresponding slopes are of −8.59 for the carbene 1 device and of 8.72 for that of carbene 2. The observed tendency evidences the photovoltaic effect on both carbene films. The Jsc values for the carbene 2 device are larger than those for carbene 1, which also can be related to the smaller gap previously reported (Table 1).

Once the carbenes capability to function as organic semiconductors has been demonstrated, PC and IPC devices were compared (see Figure 1d,e). It should be noted that the manufactured devices are not optimized, that is, the effect of thickness has not been evaluated, since the objective of this study is focused on analyzing the use of PEDOT:PSS as an anode and as an interfacial HTL. According to the experimental section (Figure 1c–e), the devices are divided into three groups: IC, PC and IPC; Table 2 shows the function performed by each component in the structure of the devices. IC and PC are unipolar devices, which consist of the carbene thin film located between the two electrodes, from which charges are injected and extracted during the electrical characterization (I-V). In order to minimize the possible current losses due to the presence of injection barriers or charge extraction barriers between the electrodes and the semiconductor, the PEDOT:PSS film was added as HTL in the IPC devices. In this way, it is expected that the inclusion of the PEDOT:PSS layer in the unipolar devices, between the anode and the carbene, will reduce the injection barrier. A more ordered and crystallized carbene film microstructure is observed when using benzoid PEDOT:PSS as nucleation layer, which improves the conduction properties and might decrease the energy barriers between the device structure components. With the above, it limits the charge extraction and also reduces the electron blocking ability which is necessary for suppressing the current leakage in the device, which is observed between PEDOT:PSS HTL with other active materials [54].

**Figure 8:**
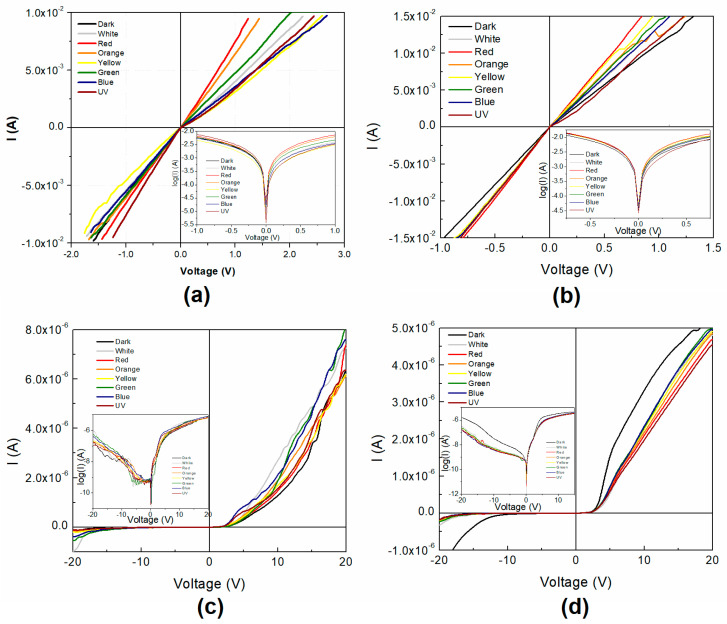
I-V characteristics and logarithmic inset of the (**a**) IC1, (**b**) IC2, (**c**) PC1 and (**d**) PC2 devices.

I-V characteristics were obtained for IC1 and IC2 while varying the incident light color, as shown in Figure 8. A logarithmic inset is included for further analysis as well. A variation of the current is observed with the variation of light colors, indicating that the device exhibits photosensitivity. The logarithmic curves for both devices show a variation of the forward and reverse bias behavior. IC2 (Figure 8b) shows current values higher than those observed for IC1 (Figure 8a), for both forward and reverse bias. Moreover, it is important to consider that the optical gap for carbene 2 is smaller than that for carbene 1, which is an indication of its better charge-carrying capacity. However, it can be stated that both materials behave as semiconductors. For these reasons, the carbene films were chosen as the active layer of all the manufactured devices. Additionally, asymmetry is observed in the curves of Figure 8a,b, which indicates a different behavior of the devices on the forward and reverse bias, hence an indication of their ambipolarity. I-V characteristics were obtained for PC1 and PC2 as shown in Figure 8c,d as a function of the incident light color. Furthermore, a logarithmic inset is included for further analysis. A variation on the current is observed with the variation of light colors, indicating that the device presents photosensitivity. By observing the logarithmic curves for both devices a variation of the forward and reverse bias behavior can be noticed. The curves observed present a typical Schottky diode behavior, which is common among devices with one organic semiconductor layer [55]. There is a clear difference between the forward and reverse operation zones. This kind of behavior arises when a Schottky barrier is formed between a semiconductor and an electrode. The absence of an ohmic contact between PEDOT:PSS, which is the electrode, and the carbene may form this barrier. There are differences between the characteristics of both carbene devices. PC1 begins to show signals of a breakdown voltage close to –20 V and its threshold voltage is 0.5 V. The measurements show some noise, especially for positive values, which can be attributed to a degradation in the device due to the presence of PEDOT:PSS, as it has been previously observed [31]. In comparison, for PC2 without illumination, the breakdown voltage is –12 V and the threshold voltage is 0.8 V. The increase on the threshold voltage observed for carbene 2 is expected, due to the increase in the activation energy. However, the current values attained by the devices decreased significantly in relation to IC1 and IC2. The values vary from around 1.5 × 10^−2^ A (1 V) to 7 × 10^−7^ A (5 V). So even when the semiconductor behavior switches to a Schottky one, the conduction could be improved. Even though the IC devices have a heterogeneous morphology and less crystallinity, the conduction is more pronounced than in the PC devices. It is important to notice that carbene 2 likewise presents higher currents than carbene 2 on the forward bias region. Diode parameters were calculated for the devices and are included in Table 3, which presents their electronic properties such as threshold voltage (V_th_), saturation current (Is), rectification ratio (RR), diode ideality factor and the slopes for the different conduction regions. Comparing the diode parameters between PC1 and PC2, it is observed that the saturation current, threshold voltage and ideality factor are larger for PC2. Calculations of the rectification ratio of the devices were obtained for 1, 15 and 20 V. It is observed that the values vary from 7.6 to 133, and from 2.7 to 104.3 for PC1 and PC2, respectively. The decrease of the RR for PC1 at 15 V is related to the current breakdown on the reverse bias observed in Figure 8c. Figure 8 also shows the light-induced photocurrent effect on PC1 and PC2, where a more pronounced effect can be observed on the logarithmic inset of Figure 8d for carbene 2. 

The forward current through the Schottky junction was determined using the following expression:

$$I=I_{s}\exp\left(\frac{\mathrm{qV}}{\mathrm{nkT}}\right)$$

where V is the applied voltage, Is is the saturation current and n is the diode ideality factor. Additionally, for the PC devices it can be divided into three conduction mechanism regions: ohmic, T-CLC and SCLC. For the ohmic and T-CLC regions the slopes are lower for PC1 compared to PC2, and more pronounced for the T-CLC region. For the SCLC region the slope is lower for PC2 (1.14) than for PC1 (2.22). The latter is because charge carriers injected from the contacts dominate the current on the I-V characteristics in the SCLC region, therefore, the current is only carrier mobility dependent and higher values yield a higher mobility, observed for PC1. Nevertheless, PC2 presents a higher conduction in the ohmic region, as well as in the T-CLC. This is indicative that PC2 may present a higher trap density than PC1. On the other hand, a small difference in n is observed from the ideal diode (*n* = 1), the latter indicating that the diodes present a nonideal behavior as a possible consequence of the interfaces and it is also increased when there is no film homogeneity.

**Figure 9:**
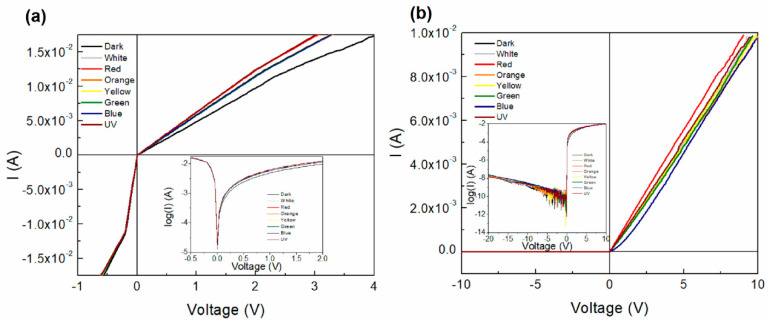
I-V characteristics of the (**a**) IPC1 and (**b**) IPC2 devices.

Comparing the IPC1 and IPC2 diode parameters (see Figure 9 and Table 3), lower values of Vth and Io are observed for IPC1. On the other hand, the ideality factor for IPC1 is 1 and 1.02 for IPC2. These values indicate that the device operation is close to the ideal diode. The RR value for IPC1 is very small compared to the previous devices, which is related to the reverse bias decay of the curve observed in Figure 9. On the other hand, the RR values for IPC2 are very large due to a very small and constant saturation current for the reverse bias compared to the forward current. In the case of the IPC devices, only two conduction mechanisms are observed, ohmic and SCLC. It is observed that IPC1 presents slightly lower values than IPC2. Compared with the PC1 and PC2 devices, it can be observed that using the PEDOT:PSS film as HTL in the devices decreases the slope in the different regions and eliminates the T-CLC mechanism.

Figure 9a shows the IPC device I-V characteristic curve that differs from what was achieved with PC1. When voltage is applied in the forward bias (Figure 9a), a linear region appears, which then saturates. However, the change from the ohmic to the saturation regions is not as abrupt as the one seen on IC1. In contrast, it seems to present a gradual change of slope beginning at 2.5 V, resembling the knee that appears in the curves of some devices [56]. Operation in the inverse bias zone presents a more pronounced ohmic curve with a marked change of slope, acting almost as a conductor until saturation is reached. As can be seen in Figure 9b, the I-V curve of IPC2 has the shape of one belonging to a diode. The logarithmic curve inset shows a more detailed device behavior on both, forward and reverse bias regions. All of the IPC2 device properties were improved in comparison with PC2. In the IPC device, where PEDOT:PSS was used as HTL, the threshold voltage is very low, around 0.1 V. The breakdown voltage was not reached even at 20 V, so further studies should be done on the thin film device in order to completely characterize it. Although this value is not known, having conduction in the forward operation zone and a small leakage current in the reverse one, is an indicator that this kind of device could be evaluated as a rectifier. Hence, the small saturation current of 1.95 × 10^−10^ A observed, at values of up to –10 V, gives evidence of a good performance of this device for such an application. Current values once again reach values of ~1.0 × 10^−2^ A in IPC2 at 10 V, while IPC1 presents higher current values of up to ~1.75 × 10^−2^ A at only 4 V. Furthermore, in Figure 9 the effect of the incident light on the I-V curves can be appreciated, which is more pronounced for the IPC1 on the forward bias region. The incorporation of benzoid PEDOT:PSS as HTL shows a better performance than as anode for carbene complexes organic solar cells, where the current is higher, the injection barrier is reduced and no T-CLC conduction mechanism is observed. Nevertheless, the Schottky curve shape is more marked for the anode function, where three conduction mechanisms are observed. Thus, the HTL has an important effect on the conductivity and photoconductivity of the devices, which in particular for carbene 1 presents an abrupt breakdown region that could allow reverse bias voltages, that was not observed for the PEDOT:PSS anode device.

## 4. Conclusions

In the present work, the behavior of PEDOT:PSS as an electrode and as a hole carrier layer was analyzed for two carbene organic solar cells. The carbene films used as active layers are more homogeneous and have a more ordered structure when they are deposited on PEDOT:PSS than on ITO. Carbene films also have a smaller optical gap on PEDOT:PSS compared to ITO, which is an indication of a better charge transport within the carbene/polymer system. Carbene 2 presents a higher activation energy than carbene 1, as well as higher conductivity and photoconductivity. A decrease of the short circuit current density against the incident light energy is observed for the carbenes and it is more pronounced for carbene 2. Small saturation currents are observed for both configurations but they are lower for IPC2. The organic solar cells present nonideal behavior as a possible consequence of the interfaces and the rather poor film homogeneity. PC1 may present higher carrier mobility than PC2, which is the opposite for the IPC devices. A light-induced photocurrent effect on the PC and IPC devices is observed and it is more pronounced for carbene 2, where PEDOT:PSS is used as anode, and for carbene 1, where it is used as HTL. The PEDOT:PSS film as HTL reduces the injection barrier and a better performance than as anode for this type of organic solar cell is observed. The current is higher for IPC than for PC, and no T-CLC conduction mechanism is observed. However, the PEDOT:PSS system without special treatment and without dopants can be applied as an electrode. Compared to ITO based structures, although the current in the device is lower, the optical bandgap has a lower value, which makes it more adequate for its use in optoelectronic devices.

## Figures and Tables

**Table 1:** Optical gap for the carbene films on ITO/Corning glass and PEDOT:PSS/Corning glass substrates.

Film	Optical Gap	Optical Gap
	(Direct Transitions)	(Indirect Transitions)
	eV	eV
carbene 1 film onto ITO	2.93	2.49
carbene 1 film onto PEDOT:PSS	2.28	2.52
carbene 2 film onto ITO	2.75	2.33
carbene 2 film onto PEDOT:PSS	2.10	2.06

**Table 2:** Function performed by each component of the devices.

Component	Function	Device
glass	Substrate	All
ITO	anode	IC1, IC2, IPC1, IPC2
PEDOT:PSS	anode	PC1, PC2
PEDOT:PSS	HTL	IPC1, IPC2
carbene 1 and 2	Active layer	All
Ag	cathode	All

**Table 3:** Electronic properties of the PC and IPC devices.

	Is (A)	Vth (V)	Io (A)	RR (1 V)	RR (10 V)	RR (15 V)	n	Slope Ohm Region	Slope T-CLC Region	Slope SCLC Region
PC1	6.13 × 10^−10^	1.33	4.35 × 10^−13^	7.63	60.13	133.03	1.35	1.50	3.66	2.22
PC2	6.39 × 10^−10^	2.02	9.68 × 10^−13^	2.77	104.39	12.24	1.83	1.89	5.42	1.14
IPC1	-------	0.27	2.46 × 10^−5^	0.16	-----	-----	1.27	1.00	-----	0.77
IPC2	1.95 × 10^−10^	0.70	3.19 × 10^−6^	1.42 × 10^7^	7.95 × 10^6^	-----	1.13	1.02	-----	1.07
